# Double contrast-enhanced ultrasound for the preoperative gross classification of gastric cancer: a comparison with multidetector computed tomography

**DOI:** 10.1186/s12880-022-00954-8

**Published:** 2022-12-21

**Authors:** Ping He, Lan Zeng, Liying Miao, Tianli Wang, Juxiang Ye, Lingmei Meng, Heng Xue, Fan Zhang, Bo Zhao, Huiyu Ge

**Affiliations:** 1 Department of Ultrasound, Peking University Third Hospital, Beijing, 100191 China; 2 Department of Ultrasound Medicine, Beijing Chaoyang Hospital, Capital Medical University, Beijing, 100020 China; 3 Department of Radiology, Peking University Third Hospital, Beijing, 100191 China; 4 Department of Pathology, Peking University Third Hospital, Beijing, 100191 China; 5 Department of Gastroenterology, Peking University Third Hospital, Beijing, 100191 China

**Keywords:** Gastric cancer, Double contrast-enhanced ultrasound, Multidetector computed tomography, Gross classification

## Abstract

**Purpose:**

To compare the diagnostic performance of double contrast-enhanced ultrasound (DCEUS) and multi-detector row computed tomography (MDCT) in the gross classification of gastric cancer (GC) preoperatively.

**Methods:**

54 patients with histology proved GC were included in this retrospective study. The sensitivity and specificity of DCEUS and MDCT for the gross classification of GC was calculated and compared. The area under the curve (AUC) from a receiver operating characteristic curve analysis was used to evaluate the difference of the diagnostic performance between these two methods.

**Results:**

There were no significant differences between DCEUS and MDCT in terms of AUC for early gastric cancer (EGC), Borrmann I, II, III and Borrmann (III + IV) (*P* = 0.248, 0.317, 0.717, 0.464 and 0.594, respectively). The accuracy of DCEUS in diagnosing EGC, Borrmann I, II and Borrmann (III + IV) was higher than that of MDCT (96% vs 92%; 96% vs 94%; 87% vs 80%; 83% vs 73%), while in determining Borrmann III and IV, that of DCEUS was lower than that of MDCT (72% vs 74%; 89% vs 96%).

**Conclusion:**

Considering the revolution in clinical decision, prognosis evaluation, safety and non-invasion aspects, DCEUS can be used as the main alternative method for Borrmann classification of GC preoperatively.

## Introduction

Gastric cancer (GC) is one of the most common cancers worldwide, the prognosis of which is closely related to the gross appearance [1–3]. According to the Japanese Gastric Cancer Association criteria, the gross appearances of GC were classified into two types: early gastric cancer (EGC) and advanced gastric cancer (AGC) [4]. For ACG, macroscopic Borrmann classification system, developed in 1926, is still a valuable clinicopathological characteristic and used by pathologists and surgeons worldwide, because it can easily be decided by macroscopic pathological examination after excision [2, 5]. The precise preoperative diagnosis and gross classification is important to the optimal treatment of GC.

Many preoperative modalities, such as multidetector computed tomography (MDCT), endoscopic ultrasound, and magnetic resonance imaging, have been used for assessing the gross classification of GC. Double contrast-enhanced ultrasound (DCEUS), in which intravenous contrast enhanced ultrasound is combined with oral contrast-enhanced ultrasound, is an accurate, well-tolerated, noninvasive diagnostic method for preoperative evaluation of GC [2, 3, 6–8]. Oral contrast-enhanced ultrasound can clearly display the stratification of gastric wall by filling the stomach with oral contrast agent, such as water. Intravenous contrast enhanced ultrasound can be used to evaluate the micro vessels and tissue perfusion, which proves to be successful in solid organs such as the liver and kidney [9, 10]. Thus, DCEUS may be a useful preoperative modality for the evaluation of the gross classification of GC.

Studies about the comparison of DCEUS and MDCT in the gross classification of GCs are limited [2]. The purpose of this study was to compare the diagnostic performance of DCEUS and MDCT in the gross classification of GCs preoperatively.

## Material and methods

### Patients

From December 2011 to January 2015, a total of 54 patients (36 men and 18 women, mean age 61 ± 9.70 years) with GC proven by endoscopic biopsy were included in this retrospective study. All patients were preoperatively examined with DCEUS and MDCT, and surgical excision was performed within a week after both examinations. This patient cohort was already published in another study, which was about the tumor staging of GC (blinded reference).

### DCEUS

The ultrasound examinations were performed using Philips iU22 system (Philips Healthcare, Bothell, WA) equipped with convex-array transducers (C5-1) and linear transducer (L9-3).

The exams were carried out after fasting for at least 6 h. Patients were asked to drink about 500–800 mL of water as quickly as possible, which was the oral contrast agent in this study that can dilate the stomach and displaces the air with in it. Within 2 min of drinking water, 2.4 mL bolus of SonoVue (Bracco SpA, Milan, Italy) was injected through antecubital vein, followed by a 5-mL saline flush. A second injection was performed if the first injection was inadequate. The waiting period between the two injections was 15 min. Then, the examination was performed with convex transducers with low mechanical index of 0.06–0.08. If possible, L9-3 transducer was used to get a better resolution. Static and dynamic images were stored and analyzed later.

### MDCT

MDCT (Somatom Definition Flash, Siemens Medical Solutions, Forchheim, Germany) was used for CT scanning. The MDCT imaging parameters were 120 kVp/250 mAs, the field of view was 100 × 100 mm2, the slice thickness was 0.6 mm, the slice interval was 0.1 mm, pitch was 0.8 and the standard matrix size used was 512 × 512 pixels.

Patients needed to fast for at least 6 h. In order to dilate the stomach, patients should drink approximately 600–1000 mL of water 5 min before CT examination. An intravenous dose of 80 mL of contrast material (ioversol, 350 mg/mL, Mallinckrodt Canada ULC, Quebec, Canada) was injected at a rate of 3 mL/s. The MDCT scans were obtained at 30 s (arterial phase) and 70 s (portal-venous phase) after intravenous contrast agent administration.

### Pathologic and image analysis

All resected specimens were examined by one of two experienced pathologists (LMM and JXY, with 11 and 8 years of experience in the field of gastroenteric tumor pathologic diagnosis), who were unaware of the DCEUS and MDCT findings. A consensus was reached by discussion in cases of disagreement. EGC was defined as a tumor limited to the mucosa or submucosa, independent of lymph node status, whereas AGC was defined as a tumor invading the muscularis propria or deeper. AGC was further classified into four growth types according to the Borrmann criteria: type I, polypoid tumor; type II, ulcerative lesion with elevated and sharply demarcated margins; type III, ulcerative lesion without definite limits, infiltrating into the surrounding gastric wall; type IV, diffusely infiltrating tumor without ulcer or a discretely marginated mass [11].

**Fig. 1:**
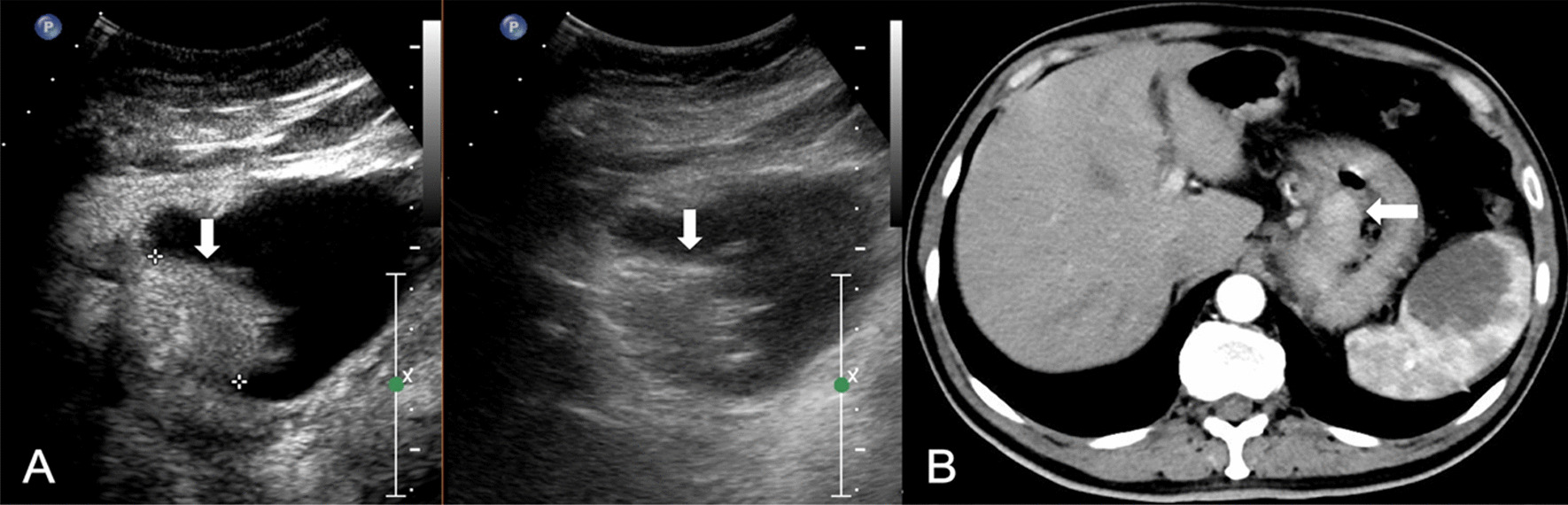
GC classified as Borrmann I on DCEUS (**A**) MDCT (**B**). The white arrow shows a polypoid tumor protruding to the gastric cavity

**Fig. 2:**
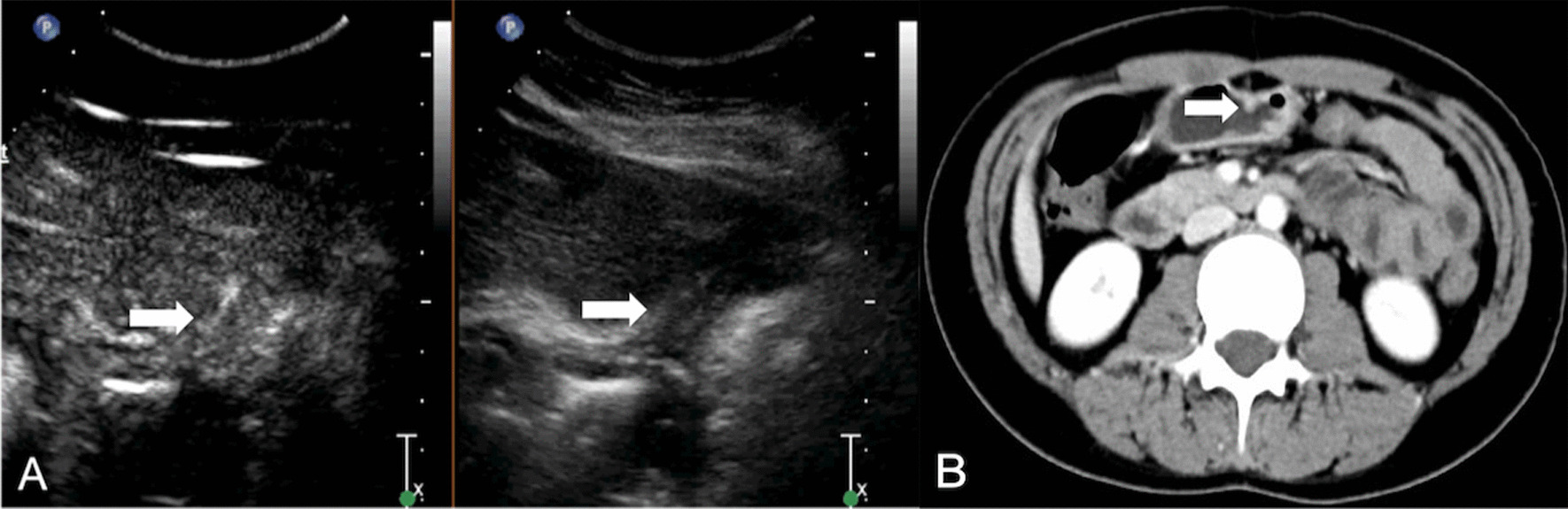
GC classified as Borrmann II on DCEUS (**A**) MDCT (**B**). The white arrow shows an ulcerative lesion with elevated and sharply demarcated margins

**Fig. 3:**
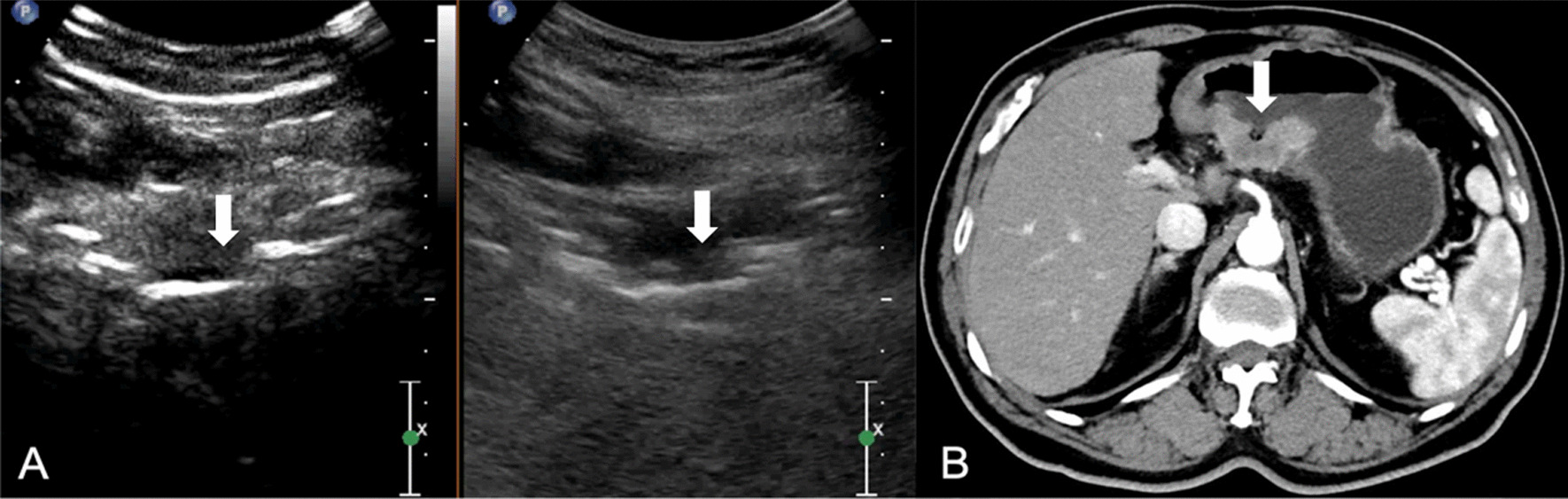
GC classified as Borrmann III on DCEUS (**A**) MDCT (**B**). The white arrow shows an ulcerative lesion without definite limits, infiltrating into the surrounding gastric wall

**Fig. 4:**
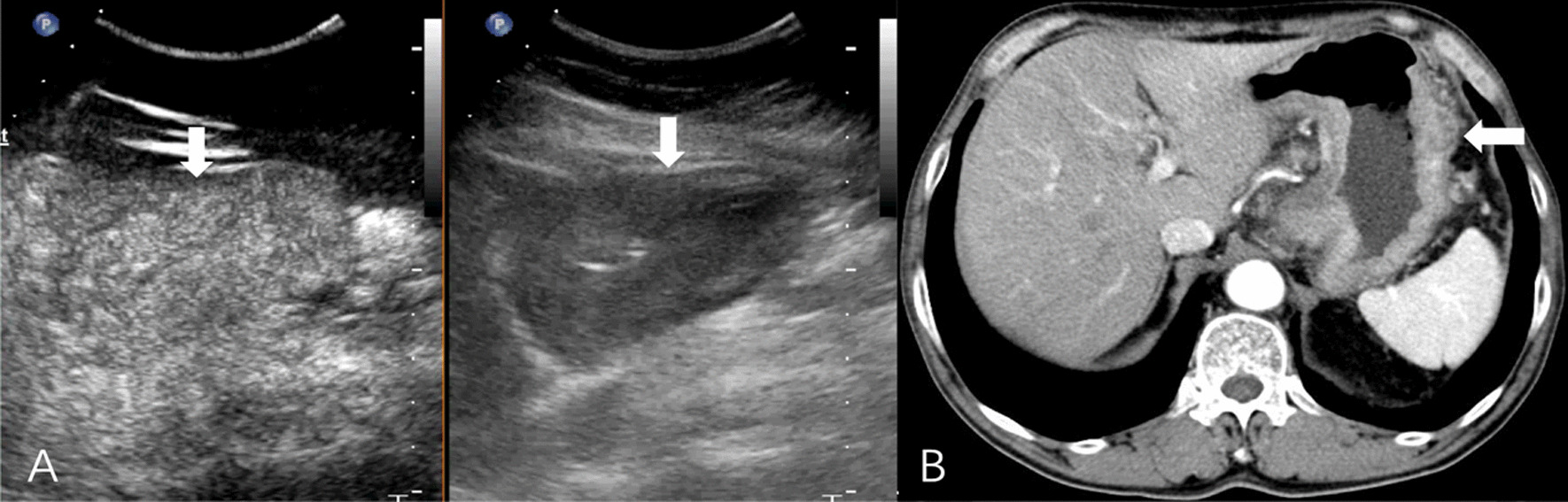
GC classified as Borrmann IV on DCEUS (**A**) MDCT (**B**). The white arrow shows a diffusely infiltrating tumor without ulcer or a discretely marginated mass

All DCEUS were examined by one of two radiologists (HYG. and LYM, with 17 and 29 years of experience in gastroenteric imaging respectively, and 14 years of experience in CEUS imaging) who were blinded to MDCT and pathological results but aware of the presence of GC. A consensus was reached by discussion in cases of disagreement. DCEUS gross classification criteria: EGC was defined as a tumor limited to the mucosa or submucosa, independent of lymph node status; type I, the tumor showed as a mass, and mainly grows into the gastric cavity (Fig. 1); type II, the section of the tumor is U-shaped with the base almost as wide as the opening of “U”, and the tumor edge rise above the surrounding gastric wall with clear boundaries (Fig. 2); type III, the section of the tumor is U-shaped with the base wider than the opening of “U”, and the edge of tumor do not rise above the surrounding gastric wall with blurred boundaries (Fig. 3); type IV, the gastric wall is diffusely thickened and stiff, with the normal wrinkles disappeared (Fig. 4).

All images of MDCT were reviewed by the same radiologist (TLW, with 30 years of experience in gastroenteric imaging), who was blinded to the CEUS findings but aware of the presence of GC. MDCT gross classification criteria: EGC was defined as a tumor limited to the mucosa or submucosa, independent of lymph node status; type I, the tumor showed as a mass, and mainly grows into the gastric cavity (Fig. 1); type II, localized thickening of the gastric wall and the depression of tumor center are observed, and the edge of tumor is higher than the gastric wall (Fig. 2); type III, the center of the tumor is depressed with the tumor mainly grows towards the base of the ulcer, and the edge of the tumor is not significantly protuberant or higher than the gastric wall (Fig. 3); type IV, the gastric wall is diffusely thickened and stiff, with the normal wrinkles disappeared (Fig. 4).

Finally, the preoperative gross classification of GC by DCEUS and MDCT was compared with the histopathologic gross classification which is the gold standard of gross classification of GC.

### Statistical analysis

The statistical analysis was performed with MedCalc version 14.8.1.0 software (MedCalc Software, Mariakerke, Belgium). Continuous variables were expressed as means ± standard deviations. The accuracy, sensitivity, specificity, and Youden’s index were calculated with DCEUS and MDCT for gross classification. The area under the curve (AUC) from a receiver operating characteristic curve analysis was used to evaluate the difference of the diagnostic performance between these two methods. For all analyses, *P* values less than 0.05 was considered statistically significant.

## Results

Fifty-four patients were finally included. Among them, the pathological classification in 8 cases was type EGC; in 2 cases was Borrmann I; in 6 cases was Borrmann II; in 30 cases was Borrmann III; in 8 cases was Borrmann IV.

Table 1 shows the accuracy, sensitivity, specificity, and Youden index of DCEUS and MDCT in determining gross classification of GC. The accuracy of DCEUS in diagnosing EGC, Borrmann I, II and Borrmann (III + IV) was higher than that of MDCT (96% vs 92%; 96% vs 94%; 87% vs 80%; 83% vs 73%), while in determining Borrmann III and IV, that of DCEUS was lower than that of MDCT (72% vs 74%; 89% vs 96%).

**Table 1:** The accuracy, sensitivity, specificity, and Youden's Index of DCEUS and MDCT for assessing gross classification of gastric cancer

	Accuracy, % (n)	Sensitivity, % (n)	Specificity, % (n)	Youden's index
*DCEUS*				
EGC	96% (52/54)	75% (6/8)	100% (46/46)	0.75
Borrmann I	96% (52/54)	100% (2/2)	96% (50/52)	0.96
Borrmann II	87% (47/54)	17% (1/6)	96% (46/48)	0.13
Borrmann III	72% (39/54)	90% (27/30)	50% (12/24)	0.40
Borrmann IV	89% (48/54)	25% (2/8)	100% (46/46)	0.25
Borrmann III + IV	83% (45/54)	92% (35/38)	63% (10/16)	0.55
*MDCT*				
EGC	92% (50/54)	62% (5/8)	98% (45/46)	0.60
Borrmann I	94% (51/54)	50% (1/2)	96% (50/52)	0.46
Borrmann II	80% (43/54)	33% (2/6)	85% (41/48)	0.18
Borrmann III	74% (40/54)	73% (22/30)	75% (18/24)	0.48
Borrmann IV	96% (52/54)	88% (7/8)	98% (45/46)	0.86
Borrmann III + IV	78% (42/54)	82% (31/38)	69% (11/16)	0.51

DCEUS, double contrast-enhanced ultrasound; EGC, early gastric cancer; MDCT, multidetector computed tomography

Table 2 reveals the AUC for each Borrmann classification of GC. There were no significant differences between DCEUS and MDCT in terms of AUC for EGC, Borrmann I, II, III and Borrmann (III + IV) (*P* = 0.248, 0.317, 0.717, 0.464 and 0.594, respectively). The AUC of MDCT for Borrmann IV was significantly higher than that of DCEUS (0.927 vs 0.625; *P* = 0.001).

**Table 2:** The areas under the ROC curves for each gross classification of gastric cancer by DCEUS and MDCT

Pathology	AUC (95%CI)		*P* value
	DCEUS	MDCT	
EGC	0.875 (0.757,0.949)	0.802 (0.671,0.898)	0.248
Borrmann I	0.981 (0.900,0.999)	0.731 (0.593,0.842)	0.317
Borrmann II	0.562 (0.421,0.697)	0.594 (0.451,0.725)	0.717
Borrmann III	0.700 (0.560,0.817)	0.742 (0.604,0.851)	0.464
Borrmann IV	0.625 (0.483,0.753)	0.927 (0.822,0.980)	0.001
Borrmann III + IV	0.773(0.618,0.928)	0.752 (0.600,0.904)	0.594

AUC, area under the curve; DCEUS, double contrast-enhanced ultrasound; CI, confidence interval; EGC, early gastric cancer; MDCT, multidetector computed tomography

## Discussion

The precise preoperative gross classification is very critical in determining the appropriate treatment for GC. Endoscopic resection was recommended as the standard strategy for EGC without submucosal infiltration [12]. Since the invasion depth AGC and Borrmann I are more superficial compared to other types of GC, patients with AGC and Borrmann I are more likely to be the candidates for endoscopic resection. Besides, Borrmann I is characterized by a large size, rare serosal invasion, lower node involvement, and location in the upper third of the stomach which should be considered in treatment and follow-up of patients with this type [13]. Borrmann III and IV have the similar clinicopathological characteristics. Li et al. reported that Borrmann classification can be used as a simple indicator of lymph node metastasis and micro margin involvement in AGC. For tumors with Borrmann III and IV, radical gastrectomy with extended lymph node dissection and sufficient proximal and distal distance from the primary tumor are more necessary. In addition, peritoneal metastasis rate in patients with Borrmann III and IV tumors was higher than other types. So, intraoperative hyperthermic peritoneal chemotherapy and postoperative peritoneal hemotherapy should be considered for patients with Borrmann III and IV tumor [14].

Furthermore, preoperative gross classification is also associated with the prognosis of GC patients. The study by Song showed that the overall survival of patients with Borrmann I and II was higher than that of patients with Borrmanni type III and IV [15]. Therefore, Borrmann imaging plays an important role in clinical decision and prognosis evaluation for patients with GC.

Pathological examination is the gold-standard for Borrmann classification of GC, but it can only get results after the surgery. MDCT is used for the preoperative evaluation of GC, but the nephrotoxicity and radiation are its limitation. DCEUS is a non-invasive, convenient and free of radiation method which can clearly show the structure of the gastric wall by oral and intravenous contrast agents.

**Fig. 5:**
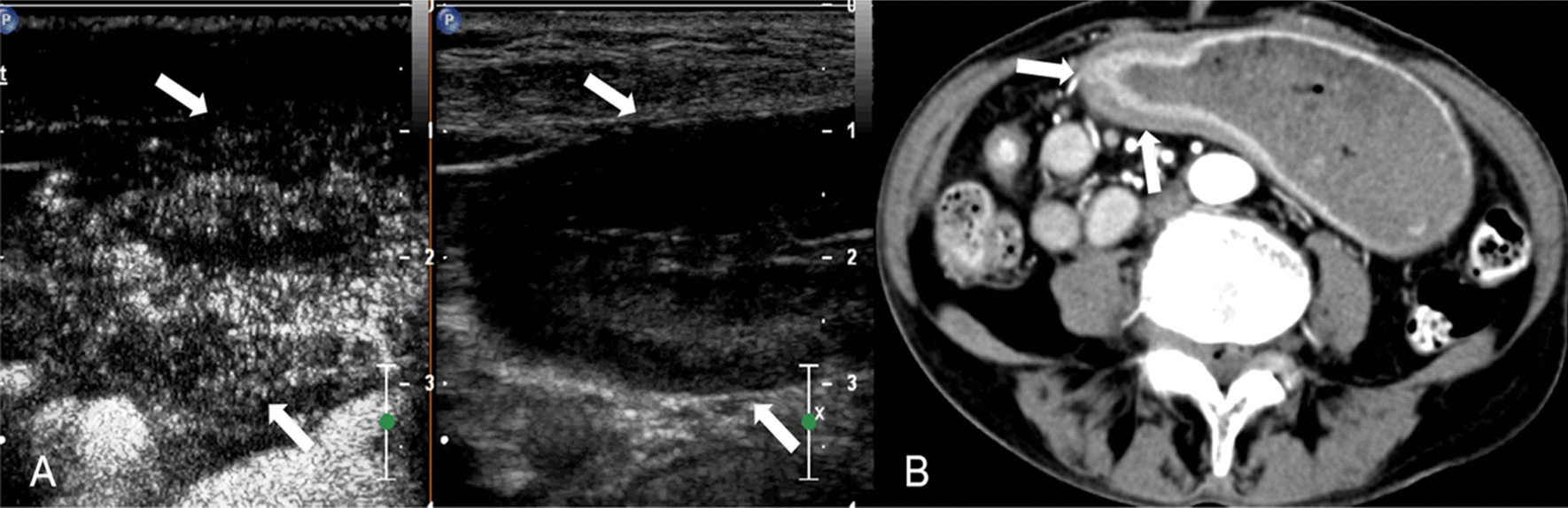
Gastric cancer classified as Borrmann IV in a 67-year-old woman by pathologic analysis. **A** An ulcerating lesion without definite limits, infiltrating into the surrounding gastric wall (white arrow) can be seen on DCEUS. Gases adhering to the surface are mistaken for ulcer and it is misdiagnosed as Borrmann III. **B** MDCT shows that diffusely infiltrating tumor without ulcer or a discretely marginated mass (white arrow), and it is classified as Borrmann IV

In this study, the diagnostic efficacy of DCEUS and MDCT were comparable in EGC, Borrmann I, II and III (all *P* > 0.05). For the diagnose of Borrmann IV, the efficacy of DCEUS was lower than that of MDCT, mainly because of the low sensitivity of DCEUS (*P* < 0.05). Interestingly, all the undetected Borrmann type IV tumors were classified as Borrmann III (Fig. 5). Considering that Borrmann III and IV have similar clinicopathological features and therapeutic strategies, we combined Borrmann III and IV into one group. And we found that the accuracy and the AUC of DCEUS in Borrmann III and IV combination was comparable to that of MDCT (*P* > 0.05). Therefore, when consider the clinical value for patients, DCEUS is an alternative choice for Borrmann classification for patients who do not want to receive radiation, be allergic to iodine and patients with kidney failure.

**Fig. 6:**
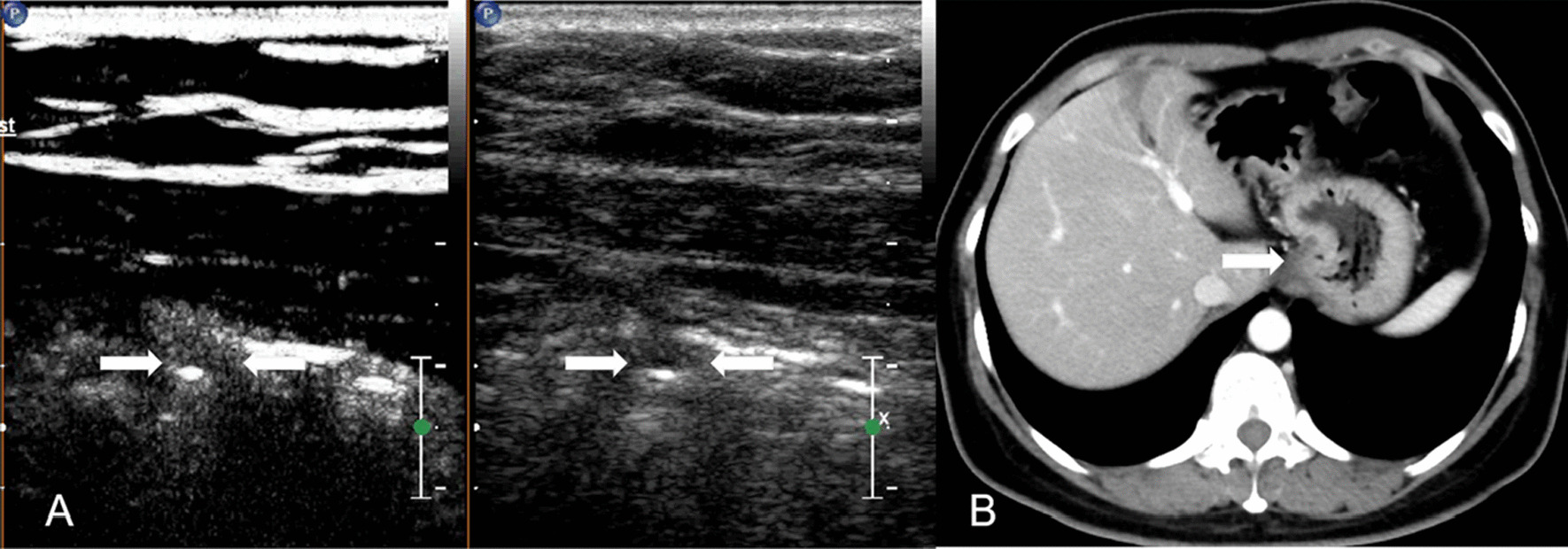
Gastric cancer classified as Borrmann I in a 41-year-old woman by pathologic analysis. **A** The thickened gastric wall with polypoid appearance (white arrows) can be seen on DCEUS, and it is classified as Borrmann I. **B** MDCT shows that thickening without infiltration into muscularis propria (white arrow), and it is misdiagnosed as EGC

The accuracy of DCEUS diagnosing EGC and Borrmann I is higher than that of MDCT, which is similar to the study reported by Yan (67% vs 57% and 87% vs 84% respectively) [2]. In this study, one case was corrected from EGC on MDCT to Borrmann I on DCEUS (Fig. 6). This is probably because DCEUS has higher spatial resolution for showing the gastric wall than MDCT and it could show five alternating hyperechoic and hypoechoic layers from inside to outside as the superficial mucosa, muscularis mucosa, submucosa, muscularis propria, and serosa [16, 17]. Thus, DCEUS is an appropriate method in determining EGC and Borrmann I for GC.

**Fig. 7:**
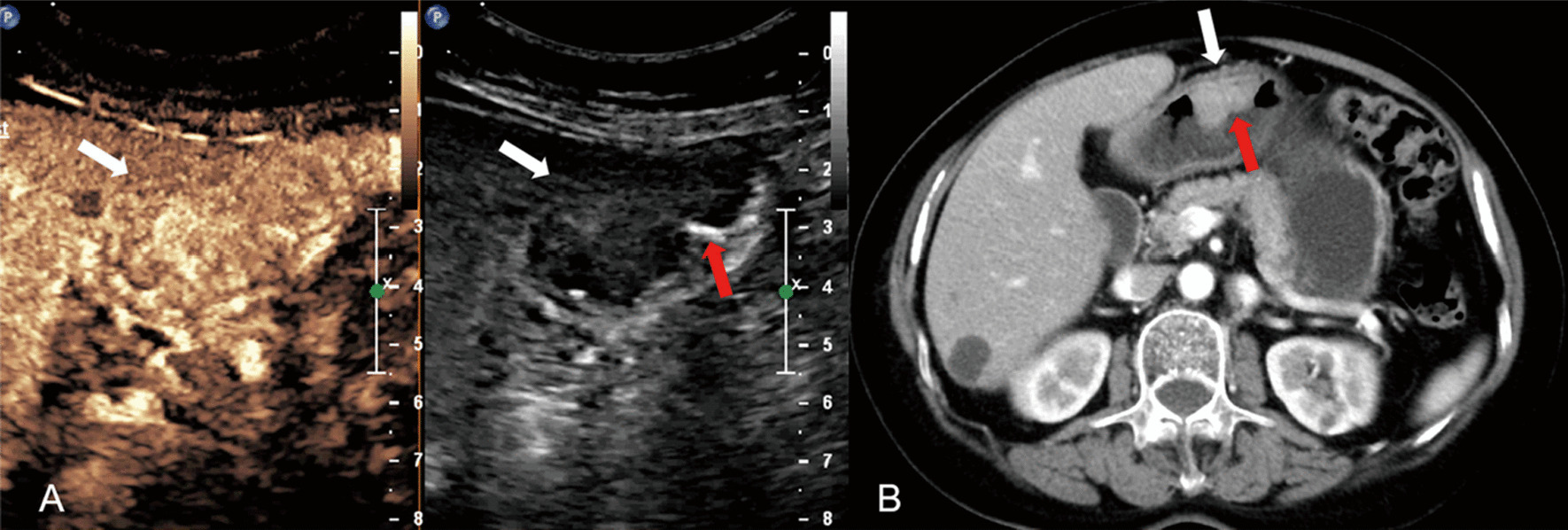
Gastric cancer classified as Borrmann II in a 70-year-old woman by pathologic analysis. **A** An ulcerating lesion (red arrow) without definite limits, infiltrating into the surrounding gastric wall (white arrow) can be seen on DCEUS, and it is misdiagnosed as Borrmann III. **B** MDCT shows an ulcerative lesion (red arrow) with elevated and sharply demarcated margins (white arrow), and it is classified as Borrmann II

Yan et al. found that both DCEUS and MDCT had high sensitivity in the diagnosis of Borrmann II (87% and 80%, respectively) [2]. And study reported by Pan also showed that the sensitivity of DCUES to diagnose Borrmann II was high (97%) [3]. However, in this study, we found the sensitivity of DCEUS and MDCT diagnosing Borrmann II was low (17% and 33%, respectively). They tended to mistake Borrmann II for Borrmann III (Fig. 7). Borrmann II is a well-bounded ulcerative lesion, while Borrmann III is an ulcerative tumor with basal invasion. To distinguish peritumor inflammation, fibrosis and tumor infiltration on DCEUS and MDCT was not easy which make Borrmann II is easily misdiagnosed. But the cases of Borrmann II is relatively small in our study. With large sample sizes, more studies can investigate the efficacy of DCUES and MDCT in distinguishing Borrmann II in the future.

There were some limitations to this study. First, this study was retrospective and only included patients referred to our hospital for surgery. GC histologically proved with biopsy was known before DCEUS and MDCT examinations. Second, although we collected data over 3 years, the number of patients who were examined with both examinations preoperatively was small in this study, especially in Borrmann I. So, the result was influenced by a sampling bias. Multicenter studies are necessary to make the results more reliable in future.

In conclusion, DCEUS has the same value in the diagnose of EGC Borrmann I, II and III, compared with MDCT. For Borrmann IV, the diagnostic efficacy of DCEUS is not as good as MDCT. However, when consider the revolution in clinical decision, prognosis evaluation, safety and non-invasion aspects, DCEUS can be used as the main alternative method for Borrmann classification of GC preoperatively.

## Data Availability

The datasets generated and analyzed during the current study are not publicly available due to the sensitive nature of the data but are available from the corresponding author on reasonable request.

## Abbreviations

GC: Gastric cancer
EGC: Early gastric cancer
AGC: Advanced gastric cancer
MDCT: Multidetector computed tomography
CEUS: Contrast-enhanced ultrasound
AUC: Area under the curve
